# Bariatric surgery in an obese patient with Albright hereditary osteodystrophy: a case report

**DOI:** 10.1186/1752-1947-7-111

**Published:** 2013-04-24

**Authors:** Chiara Ferrario, Giacomo Gastaldi, Luc Portmann, Vittorio Giusti

**Affiliations:** 1 Department of Endocrinology, University Hospital of Lausanne (CHUV), Rue du Bugnon 44, Lausanne, 1011, Switzerland

**Keywords:** Roux-en-Y gastric bypass, Pseusopseudohypoparathyroidism, Albright hereditary osteodystrophy, Bone, Calcium

## Abstract

**Introduction:**

We report for the first time the case of a patient with Albright hereditary osteodystrophy and pseudopseudohypoparathyroidism who underwent a Roux-en-Y gastric bypass.

**Case presentation:**

A 26-year-old obese Caucasian woman with Albright hereditary osteodystrophy with pseudopseudohypoparathyroidism (heterozygous mutation (L272F) in *GNAS1* exon 10 on molecular analysis) was treated with gastric bypass. She had the classical features of Albright hereditary osteodystrophy: short stature (138cm), obesity (body mass index 49.5kg/m2), bilateral shortening of the fourth and fifth metacarpals, short neck, round and wide face with bombed front and small eyes. Before the gastric bypass was performed, biochemical determination revealed a slightly low serum calcium level (2.09mmol/L; normal range 2.1 to 2.5mmol/l), and an elevated parathyroid hormone level (87ng/L; normal range 10 to 70ng/L) associated with low vitamin D level (19μg/L; normal range 30 to 50μg/L). Vitamin D supplementation was prescribed before surgery. After the Roux-en-Y gastric bypass, she achieved a progressive substantial weight loss, from 94kg (body mass index 49.5kg/m2) to 49kg (body mass index 25.9kg/m2) in one year. Her weight then stabilized at 50kg (body mass index 26kg/m2) during our three years of follow-up. Before the operation and every three months after it, she was screened for nutritional deficiencies, and serum markers of bone turnover and renal function were monitored. Considering the deficiencies in zinc, magnesium, calcium, vitamin D and vitamin B12, appropriate supplementation was prescribed. Before and two years after the Roux-en-Y gastric bypass, a dual-energy X-ray absorptiometry assessment of bone density was performed that showed no changes on her lumbar column (0.882g/cm^2^ and both T-score and Z-score of −1.5 standard deviation). In addition, bone microarchitecture with a measurement of her trabecular bone score was found to be normal.

**Conclusion:**

This is the first case of Roux-en-Y gastric bypass described in a patient with pseudopseudohypoparathyroidism showing that such a procedure seems to be safe in obese patients with Albright hereditary osteodystrophy and pseudopseudohypoparathyroidism if appropriately followed up. As obesity is a prominent feature of Albright hereditary osteodystrophy, such patients might seek bariatric surgery. After a Roux-en-Y gastric bypass, patients with Albright hereditary osteodystrophy associated with pseudopseudohypoparathyroidism need long-term follow-up on nutritional and metabolic issues.

## Introduction

Albright hereditary osteodystrophy is a disorder caused by heterozygous inactivating mutations in *GNAS*, the gene encoding the alpha chain of the stimulatory G protein and is associated with short stature, obesity, brachydactyly, subcutaneous ossifications, dental abnormalities and cognitive impairment. Because *GNAS* is paternally imprinted (silenced) in certain hormone target tissues, patients with Albright hereditary osteodystrophy with *GNAS* mutations on maternally inherited alleles often manifest resistance to multiple stimulatory G protein-coupled hormones (for example, parathyroid hormone (PTH), thyroid-stimulating hormone (TSH), luteinizing hormone (LH), follicular-stimulating hormone (FSH), and/or growth hormone-releasing hormone (GHRH)), a variant termed pseudohypoparathyroidism type 1a. The patients who inherit mutations on the paternal allele have the Albright hereditary osteodystrophy developmental defects and phenotype alone without hormonal resistance, a variant termed pseusopseudohypoparathyroidism [1]. The severity of Albright hereditary osteodystrophy phenotype is extremely variable, even among members of the same family and generation [2]. Classically, the Albright hereditary osteodystrophy obesity phenotype has been viewed as part of both conditions since first described by Fuller Albright in the mid-1950s [3]. Bariatric surgery still remains the most effective treatment for morbid obesity, leading to a reduction of comorbidities in the long term [4]. Roux-en-Y gastric bypass is one of the most frequently performed procedures in the United States and Europe. A small gastric pouch is formed, the jejunum is divided at the midsection, and the distal portion of the small intestine is anastomosed to the pouch, thus allowing nutrients to bypass the duodenum and proximal jejunum. Because these are the preferred sites for the absorption of calcium [5], patients frequently suffer from calcium and vitamin D deficiencies after Roux-en-Y-gastric bypass, which might cause secondary hyperparathyroidism [6]. Long-lasting secondary hyperparathyroidism is believed to be an important contributing factor for increased bone turnover, consequent bone loss and increased risk of osteoporotic fractures. It has been known for more than 50 years that osteomalacia and osteoporosis are frequent complications of gastric surgery and small bowel malabsorptive disorders [7]. Significant increase in serum markers of bone turnover has been documented in patients with reduced bone density after bariatric surgery [8].

We describe, for the first time, a three-year follow-up of Roux-en-Y gastric bypass in a patient with Albright hereditary osteodystrophy and pseudopseudohypoparathyroidism.

## Case presentation

This case concerns a 26-year-old Caucasian woman, known to have Albright hereditary osteodystrophy with pseudopseudohypoparathyroidism who presented herself to our department for treatment of her obesity. Her molecular analysis showed heterozygous mutation (L272F) in *GNAS1* exon 10. She had a previous history of congenital hip dysplasia, interventricular communication, strabismus, delayed growth, renal failure stage 3 and type 2 diabetes. She had never had a fracture.

**Figure 1:**
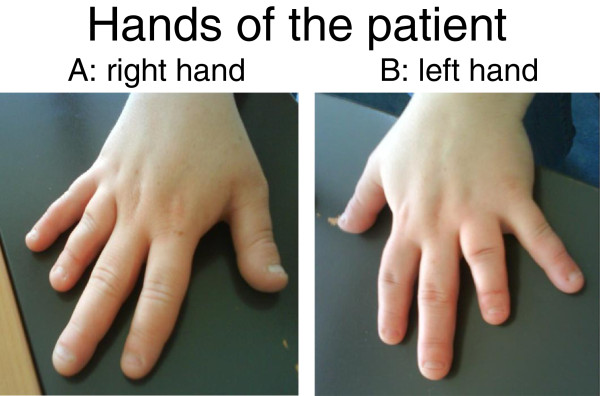
**Hands of the patient.** (**A**) right hand. (**B**) left hand.

A physical examination showed a weight of 77.7kg with a stature of 138cm (body mass index (BMI) 40.8kg/m2). She had the classical features of Albright hereditary osteodystrophy: short stature, obesity, short neck, round and wide face with bombed front and small eyes. An examination of her hands revealed a bilateral shortening of the fourth and fifth metacarpals (Figure 1).

Biochemical determination revealed normal levels of pituitary hormones TSH, human growth hormone (HGH) and insulin-like growth factor 1 (IGF-1). As she had regular menses and no signs nor symptoms of cortisol deficiency, we did not measure adrenocorticotropic hormone (ACTH), FSH and LH. She had mild hypocalcemia (2.09mmol/L; normal range 2.1 to 2.5mmol/L), low vitamin D level (19μg/L; normal range 30 to 50μg/L), and elevated PTH level (87ng/L; normal range 10 to 70ng/L), which was probably secondary to vitamin D deficiency. Phosphate and magnesium were in the normal range. Her karyotype was 46 XX.

Dual-energy X-ray absorptiometry (DEXA) assessment of bone density showed osteopenia: 0.812g/cm^2^ and both T-score and Z-score of −1.4 standard deviation (SD) on her lumbar column L1-L4. Proximal femur bone density was measured at 0.854g/cm^2^ and T-score and Z-score were both at −0.7 SD. Beta-CrossLaps were high at 869 ng/L (normal range: 25 to 573ng/L), signaling an increase in bone turnover [9]. We assessed the bone microarchitecture with a measurement of her trabecular bone score (TBS), which is a novel gray-level texture measurement that can be extracted from DEXA images and correlates with three-dimensional parameters of bone microarchitecture [10]. The TBS on the patient’s lumbar column showed a degraded skeletal microarchitecture (TBS of 1.189).

As she had hypovitaminosis D with osteopenia, vitamin D was prescribed.

As far as her obesity is concerned, her eating disorder was treated first, using group and individual psychological support, as well as dietetic and medical consultations. After two and a half years of therapy, our patient managed to improve her eating behavior and lifestyle. After a progressive weight gain, she could finally stabilize her weight at 92Kg (BMI 49.5Kg/m2). She was therefore ready to undergo a Roux-en-Y-gastric bypass.

During the first year after the procedure, she gradually achieved a substantial weight loss: from 94kg (BMI 49.5kg/m2) to 49kg (BMI 25.9kg/m2). Afterwards, her weight stabilized at 50kg (BMI 26kg/m2) during our three-year follow-up. Every three months after the Roux-en-Y gastric bypass, a screening for nutritional deficiency was performed, as well as a measurement of markers of renal function and bone turnover, as shown in Table 1. Considering the deficiencies in zinc, magnesium, calcium, vitamin D and vitamin B12, intramuscular vitamin B12 supplementation and oral zinc, magnesium, calcium and D vitamin were introduced. The doses were adjusted every three months, according to the serum vitamin level.

**Table 1:** Blood test results before and after gastric bypass

***Months after the gastric bypass***		**Pre-operative**	**3**	**7**	**10**	**15**	**18**	**24**	**30**	**33**
	***Normal range***									
Parathyroid hormone	*10 - 70 ng/L*	96	150	116	83	139	99	73	118	88
**25-OH vitamin D**_**3**_	*8.4 - 52.3 μg/L*	28.1	21.1	26.1	33.3	25.3	24	20.6	55.8	50.8
**Beta-CrossLaps**	*25 - 573 ng/L*	869	729							682

Two years after the Roux-en-Y gastric bypass, a DEXA assessment of bone density was performed, which showed no changes on her lumbar column (0.882g/cm^2^ and both T-score and Z-score of −1.5 SD), significant bone loss on her proximal femur (from 0.854g/cm^2^ to 0.754g/cm^2^ and a reduction of both T-score and Z-score from −0.7 SD to −1.5 SD). This apparent bone loss on the proximal femur is an artifact due to the significant weight loss of the patient, and consequent fat estimation error due to variation in soft tissue hydratation [9]. We assessed the bone microarchitecture with a measurement of TBS, which showed normal bone microarchitecture on her lumbar column (TBS of 1.373). It is interesting to notice a substantial improvement in her TBS, which showed a degraded skeletal microarchitecture in the pre-operative measurement and normal bone microarchitecture two years after the Roux-en-Y gastric bypass, which is partially due to the weight loss. In the analysis of TBS, obesity often leads to underestimation of the TBS score. The level of Beta-CrossLaps remained high at 682ng/L (normal range: 25 to 573ng/L), slightly lower than in the pre-operative assessment.

## Discussion

This is the first case of Roux-en-Y gastric bypass described in an obese patient with Albright hereditary osteodystrophy and pseudopseudohypoparathyroidism. Observations during the three-year follow-up shows that bariatric surgery seems to be safe in such patients. As obesity is a prominent feature of Albright hereditary osteodystrophy [3], these patients are prone to seek bariatric surgery. After a Roux-en-Y gastric bypass, such patients need long-term follow-up on nutritional and metabolic issues, as does every other patient undergoing the same surgery [11,12]. Our patient needed calcium and vitamin D supplementation before and after the surgery. A comparison in the DEXA assessments of bone density before and two years after the Roux-en-Y gastric bypass showed no changes on her lumbar column.

Bone abnormalities of Albright hereditary osteodystrophy and pseudopseudohypoparathyroidism are not well known [13,14] as calcium metabolism and skeletal health after bariatric surgery are not well known [6,15]. In our case, the elevation of PTH before and after surgery was identical, as well as the beta-crosslabs level. Further long-term prospective studies are critical to confirm if bariatric surgery in obese patients with Albright hereditary osteodystrophy and pseudopseudohypoparathyroidism is indeed safe.

## Conclusion

We present the first published case of Roux-en-Y gastric bypass in a patient with pseudopseudohypoparathyroidism showing that such a procedure seems to be safe in obese patients with Albright hereditary osteodystrophy and pseudopseudohypoparathyroidism if appropriately followed up. Obesity is a prominent feature of Albright hereditary osteodystrophy, and such patients may seek bariatric surgery. After a Roux-en-Y gastric bypass, patients with Albright hereditary osteodystrophy associated with pseudopseudohypoparathyroidism need long-term follow-up for nutritional and metabolic issues.

## Consent

Written informed consent was obtained from the patient for publication of this case report and accompanying images. A copy of the written consent is available for review by the Editor-in-Chief of this journal.

## Competing interests

The authors declare that they have no competing interests.

## Authors’ contributions

GG managed the patient in the pre-operative period and during the first months after the gastric bypass. CF managed the patient after the gastric bypass. She reported the initial clinical presentation, the diagnostic tests before and after the operation and the follow-up. She was the main contributor to this manuscript. LP and VG reviewed the manuscript and contributed to the analysis of the literature. All authors read and approved the final manuscript.
